# Targeting SIK2 with GRN-300 Potentiates Paclitaxel Efficacy in Triple-Negative Breast Cancer

**DOI:** 10.3390/cancers18111843

**Published:** 2026-06-04

**Authors:** Marc A. Pina, Rumeysa Ozyurt, Weiqun Mao, Hailing Yang, Janice M. Santiago-O’Farrill, Zhen Lu, Robert C. Bast

**Affiliations:** Department of Experimental Therapeutics, the University of Texas MD Anderson Cancer Center, Houston, TX 77054, USA; mapina@mdanderson.org (M.A.P.); rozyurt@mdanderson.org (R.O.); wmao@mdanderson.org (W.M.); hyang3@mdanderson.org (H.Y.); jmsantiago@mdanderson.org (J.M.S.-O.)

**Keywords:** TNBC, GRN-300, SIK2, paclitaxel, cell cycle, CDC27

## Abstract

Background/Objectives. Breast cancer is the most frequently diagnosed cancer worldwide, with approximately 15% classified as Triple-Negative Breast Cancer (TNBC). TNBC is characterized by the absence of estrogen receptor (ER) and progesterone receptor (PR), and the lack of HER2 overexpression, limiting use of targeted therapies. Current TNBC treatment relies heavily on chemotherapy, most commonly taxanes including paclitaxel that stabilize microtubules, disrupt chromosome separation and induce apoptosis. TNBCs frequently develop chemoresistance after multiple treatment cycles, highlighting a critical unmet need for novel therapeutic strategies. This study addresses this challenge by targeting salt-inducible kinase 2 (SIK2), which is overexpressed in 85% of TNBCs compared to normal breast tissue. Methodes. In collaboration with Arrien Pharmaceuticals and Greenfire Biologics, we developed ARN-3261/GRN-300, a novel orally bioavailable SIK2 inhibitor and evaluated its ability to sensitize TNBC cells to paclitaxel in vitro and in vivo. Results. GRN-300 demonstrated strong synergy with paclitaxel in all eight TNBC cell lines tested, as indicated by favorable combination indices. In xenograft models, the combination therapy significantly enhanced tumor growth inhibition and prolonged survival compared to either agent alone. Mechanistic studies showed that GRN-300 disrupts the anaphase-promoting complex/cyclosome (APC/C) pathway by downregulating key mitotic regulators, including CDC27, CDK1, and PLK1, thereby potentiating G2/M cell cycle arrest and apoptosis. Conclusions. Together, these findings establish GRN-300 as a promising therapeutic agent that enhances paclitaxel efficacy through complementary disruption of mitotic regulatory pathways, providing strong preclinical rationale for clinical development in TNBC.

## Simple Summary

Triple-Negative Breast Cancer (TNBC) is an aggressive subtype that lacks key therapeutic targets, leaving chemotherapy, particularly paclitaxel as a primary treatment option. This study targets salt-inducible kinase 2 (SIK2), which is overexpressed in most TNBCs. In collaboration with Arrien Pharmaceuticals and Greenfire Bio, we developed ARN-3261/GRN-300, an orally bioavailable SIK2 inhibitor, and evaluated its ability to enhance paclitaxel efficacy. Across multiple TNBC cell lines and xenograft models, the combination therapy demonstrated strong synergy, leading to greater tumor growth inhibition and prolonged survival compared to either agent alone. Mechanistically, GRN-300 disrupts mitotic regulation by downregulating key components of the anaphase-promoting complex/cyclosome pathway, including CDC27, CDK1, and PLK1, thereby promoting G2/M arrest and apoptosis. These findings support SIK2 inhibition as a promising strategy to enhance chemotherapy response and overcome resistance, providing a strong rationale for clinical development in TNBC.

## 1. Introduction

Breast cancer remains one of the most frequently diagnosed malignancies in women worldwide [1]. With continued advances in early detection and screening, the incidence has increased approximately 1% annually [2]. In the United States in 2024, an estimated 310,720 new breast cancer cases were diagnosed in women, representing over 30% of all female cancer diagnoses [3]. Many breast cancers express several protein receptors that serve as therapeutic targets, including estrogen receptor (ER), progesterone receptor (PR) and human epidermal growth factor receptor 2 (HER2). Approximately 15% of breast cancers lack ER and PR and do not overexpress HER2, defining Triple-Negative Breast Cancer (TNBC) [4,5]. TNBCs represent one of the most aggressive, highly metastatic, and heterogeneous breast cancer subtypes, characterized by higher mortality rates compared to the other subtypes. This poor prognosis relates, in part, to the absence of targetable hormone receptors and HER2, limiting therapeutic options. Unlike other breast cancer subtypes amenable to targeted therapies, TNBC treatment relies primarily on systemic chemotherapy. Currently, the FDA has approved treatment strategies for TNBC including taxane-based chemotherapy (paclitaxel and docetaxel), platinum derivatives (carboplatin), anthracyclines (doxorubicin), alkylating agents (cyclophosphamide), targeted therapy (PARP-inhibitors and antibody-drug conjugate therapy) and immunotherapy (pembrolizumab and other immune checkpoint inhibitors) [6,7,8,9,10,11]. Paclitaxel, a microtubule-stabilizing agent, is the most commonly used chemotherapeutic for TNBC and is FDA-approved as a first-line therapy for multiple malignancies, including ovarian, lung, and pancreatic cancers, as well as hormone receptor-positive breast cancer and metastatic TNBC [12]. Paclitaxel promotes microtubule assembly and stabilizes tubulin subunit associations, leading to mitotic arrest at the spindle assembly checkpoint (SAC). This prolonged mitotic delay ultimately triggers aberrant cell division and apoptosis [12,13]. Despite its widespread use, not all TNBCs respond to paclitaxel, and treatment outcomes remain suboptimal. The prognosis for metastatic TNBC is particularly poor, with a median overall survival of approximately 13.6 months [14]. Although paclitaxel improves outcomes in early-stage breast cancer, TNBC patients receiving neoadjuvant chemotherapy experience significantly shorter progression-free survival (PFS) compared to those with other breast cancer subtypes (63% versus 76%) [14]. These observations underscore an urgent need for strategies to enhance paclitaxel efficacy in TNBC [15,16].

One promising strategy to enhance paclitaxel efficacy is combination therapy with an inhibitor of salt-inducible kinase 2 (SIK2), a serine threonine kinase and a member of the AMPK family. SIK2 regulates diverse cellular processes, including the Hippo, PI3K-AKT, and Wnt/β-Catenin pathways, while also regulating metabolic homeostasis and centrosome dynamics [1,17,18,19,20]. Given its involvement in these critical pathways, SIK2 has emerged as a potential therapeutic target in ovarian cancer and TNBC [21,22]. GRN-300 is a potent selective small molecule inhibitor of SIK2. Previous studies demonstrated that GRN-300 treatment reduces cancer cell and xenograft growth and enhances sensitivity to paclitaxel in ovarian cancer models [22]. Moreover, SIK2 localizes to kinetochores and mitotic spindles during cell division, particularly in *FANCA*-deficient cells. SIK2 inhibition by ARN-3236, a small molecule closely related to GRN-300, impaired SAC function and caused cytokinetic defects, resulting in hyperploidy and polynucleation [23]. These findings suggest that SIK2 inhibition may enhance paclitaxel efficacy by disrupting mitotic regulation through complementary mechanisms. Given the urgent need for novel combination therapies for TNBC and the emergence of chemoresistance to standard chemotherapy, we investigated whether the SIK2 inhibitor GRN-300 could enhance paclitaxel-induced cytotoxicity in TNBC models. We also sought to elucidate the molecular mechanism(s) underlying this synergy and to define SIK2’s role in mitotic regulation and cell division.

## 2. Materials and Methods

### 2.1. Immunohistochemical (IHC) Staining

Tumor microarray slides (BR1301 and BR1401, TissueArray, Derwood, MD, USA) were deparaffinized using xylene and rehydrated through a series of graded ethanol solutions. Antigen retrieval was performed in Tris-based Antigen Unmasking Solution (H-3301-250, Vector Laboratories, Newark, CA, USA) at 95 °C for 10 min (min). Slides were then subjected to sequential blocking steps: PeroxAbolish (PXA969M, BioCare Medical, Pacheco, CA, USA) for 30 min, Rodent Block M (RBM961, BioCare Medical) for 1 h, and 3% Bovine Serum Albumin (BSA) in Phosphate-Buffered Saline (PBS) for 30 min. For SIK2 protein detection, slides were incubated overnight at 4 °C with a primary antibody (636702, BioLegend, San Diego, CA, USA) diluted 1:30 in 3% BSA in PBS, followed by VisUCyte HRP Polymer (DB801, R&D Systems, Minneapolis, MN, USA). Images were acquired using an Olympus IX71 microscope equipped with a DP74 camera (Evident Scientific, Webster, TX, USA). Staining intensity and scoring were independently evaluated by two pathologists in a blinded manner.

### 2.2. Cell Lines and Culture Conditions

Human breast cancer cell lines (MDA-MB-231 (MB-231), MDA-MB-436 (MB-436), MDA-MB-468 (MB-468), MDA-MB-453 (MB-453), SUM159, HCC1143, HCC1395, HCC1428, HCC1569, HCC1937, HCC1954, HCC202, HCC38, HCC70, BT-474, T47D, MCF7, SKBr3, and ZR75-1) were obtained from ATCC (Manassas, VA, USA) and Cal51 was purchased from Creative Bioarray (Shirley, NY, USA). KPL-4 was maintained in Dr. Bast’s laboratory; the original source could not be verified. (Table S1 for additional cell line information). Cell lines were cultured in either RPMI-1640 (Corning) or DMEM (Corning, Corning, NY, USA) media supplemented with 10% FBS (GeminiBio, West Sacramento, CA, USA), 200 mM L-Glutamine, and 100 mM Sodium Pyruvate (Corning). SUM159 cells were cultured in Ham’s F12 media supplemented with 10%FBS, insulin (Thermo Fisher Scientific, Sugar Land, TX, USA), 1X hydrocortisone (Thermo Fisher Scientific), and 4-(2-hydroxyethyl)-1-piperazineethanesulfonic acid (HEPES) (Thermo Fisher Scientific). All cell lines were maintained at 37 °C in a humidified atmosphere with 5% CO_2_ and were regularly tested for mycoplasma contamination.

### 2.3. Paclitaxel and GRN-300 Cytotoxicity Assays

Cell viability was assessed using the Cell Titer-Glo (Promega, Madison, WI, USA) luminescent cell viability assay. The paclitaxel IC_50_ value was obtained by seeding 2000–4000 cells per well, depending on doubling time, onto a 96-well plate (Corning) and treated 24 h after seeding using serial dilutions of paclitaxel with 100 nM being the highest dose. Cancer cells were incubated for 72 h at 37 °C in 5% CO_2_. Then the plate’s media was removed carefully from wells to avoid disturbing the cells, and 30 µL of Blank Media + 30 μL of Cell Titer-Glo reagent was added to each well. The plates were shaken at 600 rpm for 15 min in the dark, and luminescence was measured using a Synergy 2 microplate reader (Agilent Bio-Tek, Winooski, WT, USA). For combination treatment with both paclitaxel and GRN-300, cells were seeded at 2000–4000 cells per well (density adjusted based on doubling time) in 96-well plates and treated with serial dilutions of paclitaxel (0 nM to the cell line-specific IC_50_) and GRN-300 (0–2 µM). Paclitaxel was administered once at 24 h post-seeding. GRN-300 was administered at both 24 h and 48 h post-seeding. The plates were incubated at 37 °C in 5% CO_2_ for 72 h total. Then, blank media and cell Titer-glo were added as described above. The plates were shaken, and luminescence was measured using a microplate reader. Raw values were normalized to control wells using Microsoft Excel and graphed using GraphPad Prism 10. Combination index (CI) values were calculated using CalcuSyn Version 2.11 (Biosoft, Cambridge, UK).

### 2.4. Clonogenic Assays

Cells were harvested from culture plates using 0.25% trypsin in RPMI or Ham’s medium with 2.21 mM EDTA and 1X sodium bicarbonate (Corning), then seeded at a density of 600 cells per well in 12-well plates (Corning). Cells were allowed to adhere to the plate for 48 h, before removing media and providing their respective growth mediums. GRN-300 was added to achieve a concentration of 0.5 μM, 1 µM, or 2 μM per well. Plates were incubated at 37 °C in 5% CO_2_ for 24 h before GRN-300 was added for a second time. When cell growth had achieved 85% confluency, medium was removed, and wells were washed with 1 mL of PBS and stained with 1 mL of 0.5% crystal violet dye for 10 min. Plates were then washed until excess dye was removed from all wells and allowed to dry overnight protected from light. On the next day, plates were imaged using a ChemiDoc MP Imaging System (Bio-Rad Laboratories, Hercules, CA, USA). Colony area was analyzed using ImageJ software (open-source software).

### 2.5. Cell Cycle Analysis

Breast cancer cells were harvested with 0.25% trypsin and seeded onto 100 × 20 mm dishes (Corning) at 5.0 × 10^5^ cells per dish. Cells were allowed to adhere to the plate overnight and after 24 h, medium was replaced with fresh medium containing either vehicle control, GRN-300 (1 µM), paclitaxel (2 nM), or both GRN-300 (1 µM) and paclitaxel (2 nM). Paclitaxel was administered 24 h post-seeding, while GRN-300 was administered at, 24 h and 48 h, post-seeding. Cells and media were collected at 24 h, 48 h, and 72 h post-treatment. After centrifugation, the supernatant was removed, and cells were fixed by adding 5 mL of 70% cold ethanol dropwise to tubes while vortexing. Fixed cells were stored overnight at 4 °C. Cells were pelleted by centrifugation, the supernatant was removed, cells were washed once in PBS before stained with FX Cycle PI/RNase staining solution (Thermo Fisher Scientific) at room temperature and protected from light for 30 min. Samples were analyzed using Gallios flow cytometer (Beckman Coulter, Brea, CA, USA) with assistance from the UT MD Anderson Cancer Center (MDACC) South Campus Flow Core. Flow cytometry data files were analyzed using the FlowJo program (Waters Biosciences, Ashland, OR, USA). Debris was excluded by gating the cell population using FS-H vs. SS-H axis followed by FS-A vs. FS-H gating to select single cells. Cell cycle distribution was determined from the FL3-A PI histograms, and the percentage of G1, S and G2 populations were quantified.

### 2.6. Annexin V-FITC Assay

Breast cancer cells were harvested with 0.25% trypsin and seeded onto 100 × 20 mm dishes (Corning) at 5.0 × 10^5^ cells per dish. Cells were allowed to adhere to the plate overnight and after 24 h, the medium was replaced with fresh medium containing either vehicle control or GRN-300 (1 µM or 2 μM). GRN-300 was administered twice, 24 h and 48 h, post-seeding. After 48 h of GRN-300 treatment, both the medium and cells were collected via trypsinization and centrifuged at 1500 rpm for 5 min. The supernatant was removed and cells were washed twice with 5 mL of PBS, resuspended in 1X Annexin V-binding buffer and stained with 100 µg/mL Propidium Iodide (PI) mixed with and FITC-Annexin V (A13201, Thermo Fisher Scientific). Cells were incubated for 15 min at room temperature covered from light. With the assistance of the MDACC South Campus Flow Core Laboratory, fluorescence was measured at 495/519 excitation/emission using a Fortessa X-20 analyzer (Waters Biosciences).

### 2.7. Western Blot Analysis

Breast cancer cells were cultured with diluent, GRN-300, paclitaxel or the combination. After 24 h, 48 h, and 72 h, cells were collected and treated with lysis buffer (50 mM HEPES pH 7.0, 150 mM NaCl, 1.5 mM MgCl_2,_ 1 mM EGTA, 10% glycerol, 1% Triton X-100, 50 mM NaF, 1 mM Na_3_VO_4_, 1 mM PMSF, 10 μg/mL leupeptin, and 10 μg/mL aprotinin) on ice for 40 min, vertexing every 10 min. Lysates were then centrifuged at 14,800 rpm for 30 min at 4 °C. The supernatant was transferred to a clean Eppendorf tube. The protein concentration of each sample was determined with a bicinchoninic acid protein assay (23225, Thermo Fisher Scientific). Equal amounts of protein were loaded onto a 4–15% gradient Criterion^TM^ TGX^TM^ precast gel (5671085, Bio-Rad) and electrophoresed in 1X Running Buffer (10X Tris/Glycine/SDS; 1610772, Bio-Rad) at 120 volts for 1–2 h. They were then transferred to 0.45 μm PVDF membranes (IPVH00010, Millipore Sigma, Burlington, MS, USA) in 1X Transfer Buffer (10X Tris/Glycine Buffer; 1610771, Bio-Rad) at 35 volts overnight at 4 °C. The membranes were blocked with 2% BSA (A7906, Millipore Sigma) in 1X TRIS-Buffered Saline (7732-18-5, Research Products International, Mount Prospect, IL, USA) with 0.1% Tween-20 (Tween201, MP Biomedical, distributed by Thermo Fisher Scientific) and incubated with primary antibodies at 4 °C overnight. Following this, 1:2000 HRP-conjugated secondary antibody (31439, goat anti-mouse and 31463, goat anti-rabbit from Thermo Fisher Scientific) was added to the membranes while rocking at room temperature for 60 min. The protein bands were visualized using Pierce ECL Western Blotting Substrate (32106, Thermo Fisher Scientific) on a ChemiDoc MP Imaging System (Bio-Rad). Primary antibodies used in this study include: SIK2 (6919), CDK1 (9116), phospho-CDK1 T14 (2543), PLK1 (4513), phospho-PLK1 T210 (5472), phospho-Histone H2A.X (2577) and cleaved Caspase-3 (9661) from Cell Signaling Technology, Danvers, MA, USA; Cyclin B1 (sc-7393), CDC27 (sc-9972) and Lamin B1 (sc-365214) from Santa Cruz Biotechnology, Dallas, TX, USA; phospho-CDC27 (600-401-866) from Rockland Immunochemicals, Pottstown, PA, USA; and alpha-Tubulin (T7451) and GAPDH (MAB374) from Millipore Sigma.

### 2.8. SIK2 siRNA Knockdown

Human SIK2 (Gene ID: 23235) siRNA—SMARTpool, 10 nmole was purchased from Horizon Discovery Dharmacon Reagents, Lafayette, CO, USA, (Ref# SO-3278441G). Pool siRNA target sequences were as follows: GGUAUGUCCUGGUGAAUUA, CAAGAGCUAUAACCACUUU, GGACCGACUCUUCCAAUUU, and GGUGUGUGCUAUUGCAUAU. SUM159 and MB-231 breast cancer cells were seeded onto a 6-well plate at a density of 50,000 cells per well. Twenty-four hrs post-seeding, the desired siRNA concentrations were diluted in serum free media. This mixture was incubated at room temperature for 5 min before adding to the cells. SUM159 was transfected with 20 nM of siRNA and MB-231 was transfected with 35 nM of siRNA in serum free media. Plates were incubated for 72 h, before adherent cells were harvested with trypsin. Cells were centrifuged at 1500 rpm for 5 min, supernatant was removed, and cell pellets were lysed in RIPA buffer supplemented with protease and phosphatase inhibitor (PPI). Following sample preparation as described above, proteins were analyzed by Western blot using antibodies against SIK2, CDC27, phospho-CDC27, CDK1, PLK1, and GAPDH.

### 2.9. MDA-MB-231 and SUM159 Breast Cancer Xenografts

Forty female nu/nu mice (6–8 weeks old) were injected with 3.0 × 10^6^ cells into their fourth mammary fat pads. After 14 days, mice were randomly assigned to 4 groups (n = 10): vehicle control, GRN-300 (50 mg/kg; 5 times per week), Paclitaxel (8 mg/kg; once a week), and a combination of GRN-300 and paclitaxel. GRN-300 was administered by oral gavage and paclitaxel was administered by intraperitoneal injection (ip). Tumors were measured weekly and monitored until tumors grew beyond an ethically acceptable endpoint, at which point, the animals were euthanized. Animals were purchased from Inotiv, Lafayette, IN, USA. All animal experiments were approved by the Institutional Animal Care and Use Committee (IACUC) and conducted in accordance with institutional guidelines.

### 2.10. Virus Generation and Infection

The lentivirus pLOC-CDC27 (Horizon Discovery Dharmacon Reagents Clone ID: PLOHS_100005410) was obtained from the MDACC shRNA and ORFeome Core Facility. MB-231 and SUM159 were seeded onto 6-well plates at a density of 100,000 cells per well in 2 mL of respective media. After allowing the cells to attach to the plate for 24 h, cells were transduced at an MOI of 10. The stock pLOC-CDC27 lentivirus concentration was 2 × 10^6^ TU/mL and the stock pLOC-RFP vector concentration was >10^8^ TU/mL. Additionally, polybrene was added to assist with transduction efficiency at a dilution of 1:1000. After an additional 48 h, cells were treated with blasticidin antibiotic selection agent at a concentration of 8 µg/mL.

### 2.11. Statistical Analyses

Data was compared using Student *t*-test, one-way ANOVA (Analysis of Variance), or two-way ANOVA. In vivo tumor growth was analyzed using two-way ANOVA and presented as mean ± SEM (Standard Error of the Mean). Survival analysis was performed with Kaplan–Meier survival curves using a log-rank test. Statistical analyses and graphical representations were performed using GraphPad Prism 10 (GraphPad Software; San Diego, CA, USA). A *p*-value < 0.05 was considered statistically significant.

## 3. Results

### 3.1. SIK2 Is Highly Expressed in Clinical TNBC Clinical Specimens and Cell Lines

**Figure 1:**
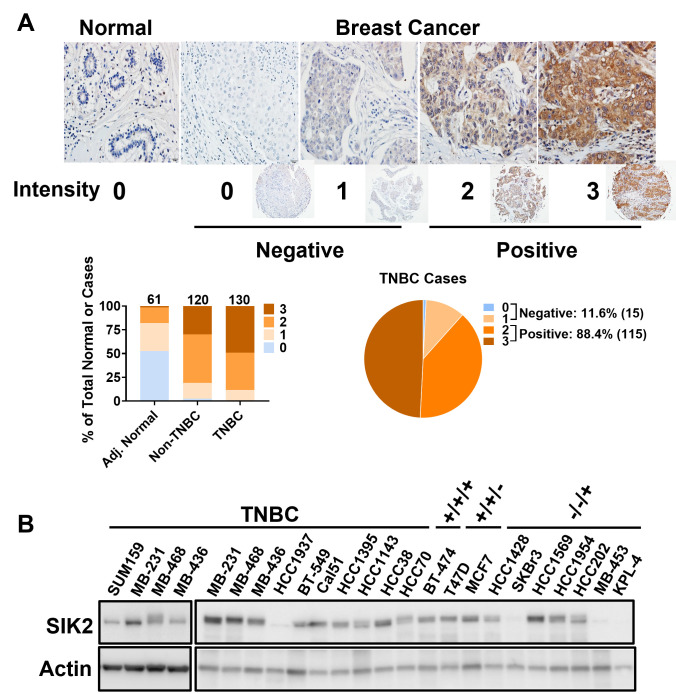
(**A**) Tissue microarray tissue samples stained with anti-SIK2 antibody. Stain intensity was measured in 250 samples and intensities was given a grade ranging from 0 to 3. 0–1: Negative; 2–3: Positive. (**B**) Western blots of SIK2 expression across multiple breast cancer cell line types. SUM159 cells and three representative TNBC cell lines were analyzed for SIK2 expression in a separate gel (first four lanes). Estrogen receptor (ER); Progesterone receptor (PR); HER2 positive (+/+/+); ER/PR positive but HER2 negative (+/+/−); ER/PR negative but HER2 positive (−/−/+).

To evaluate SIK2 expression in normal tissue and in breast cancers, we performed IHC analysis using a validated anti-SIK2 antibody (Figure 1A). Across 250 breast cancer specimens, approximately 85% exhibited some SIK2 expression. Notably, SIK2 expression was elevated in TNBC, with 88% of the 130 TNBC cases, demonstrating intense cytoplasmic staining. In contrast, non-TNBC specimens (n = 120) exhibited intense staining in only 30% (Figure 1A), suggesting that SIK2 expression is enriched in the TNBC subtype. Consistent with these findings, Western blot analysis of a broad panel of breast cancer cell lines showed detectable SIK2 expression in 9/10 TNBC cell lines, with generally higher expression levels compared with other breast cancer subtypes (Figure 1B). We further evaluated SIK2 expression in the TNBC cell line SUM159. Western blot analysis of SUM159 together with three representative TNBC cell lines was performed in a separate panel, confirming modest SIK2 expression in SUM159 cells (Figure 1B). Together, these data demonstrate a strong concordance between SIK2 expression patterns in primary TNBC specimens and TNBC cell lines, supporting the use of these models for subsequent mechanistic studies.

### 3.2. GRN-300 Enhances Paclitaxel Sensitivity and Synergistic Cytotoxicity in TNBC Cell Lines

**Figure 2:**
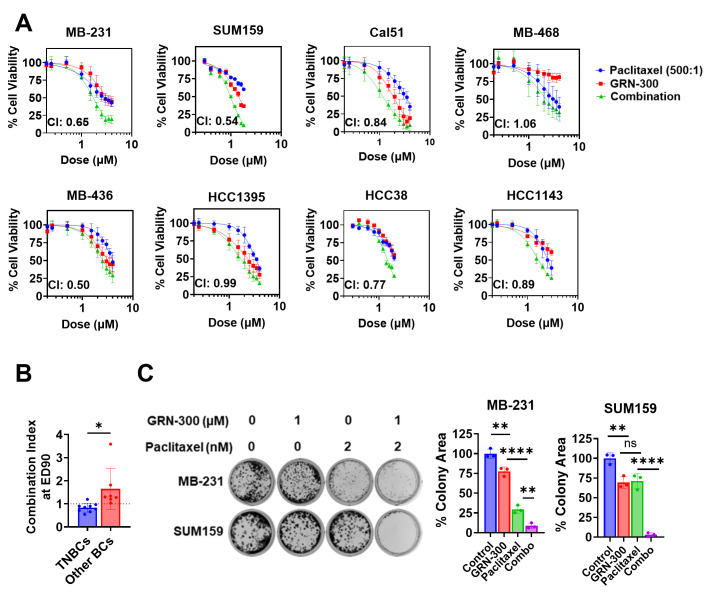
(**A**) The analysis of combination treatment in TNBC cell lines. Paclitaxel IC_50_ values were independently determined for each cell line using GraphPad Prism 10 and used to guide combination treatment with GRN-300. Dose–response curves were generated using GraphPad Prism 10. (**B**) Combination index (CI) at ED90 calculated using CalcuSyn software. CI values were compared between TNBC cell lines and non-TNBC breast cancer cell lines and plotted using GraphPad Prism 10. Statistical significance was determined by *t*-test. *, *p* < 0.05. (**C**) Clonogenic assay of MB-231 and SUM159 cells lines treated with GRN-300 alone, paclitaxel alone, or the combination. Colonies were stained with 0.5% crystal violet. Colony area was quantified using ImageJ, and statistical significance was determined by one-way ANOVA using GraphPad Prism 10. ns, *p* > 0.05, ** *p* < 0.01, **** *p* < 0.0001.

We next assessed whether SIK2 inhibition could potentiate paclitaxel activity in TNBC. First, we established the intrinsic sensitivity of each TNBC cell line to paclitaxel (0–100 nM) using CellTiter-Glo viability assays and determined the individual IC_50_ values, which ranged widely from 2 nM to 61 nM, reflecting the heterogeneous drug responses across the TNBC panel. We then examined whether GRN-300 could enhance paclitaxel efficacy at sub-IC_50_ concentrations. Co-treatment experiments using serial dilutions of GRN-300 (0–2 μM) and paclitaxel demonstrated that all eight TNBC cell lines tested exhibited significantly increased sensitivity to the combination relative to either agent alone (Figure 2A). In contrast, none of the seven non-TNBC cell lines showed meaningful enhancement (Figure S1), suggesting that the GRN-300-paclitaxel interaction is TNBC-selective. Combination index (CI) analysis using CalcuSyn revealed synergistic interactions (CI < 1) in the majority of TNBC cell lines (Figure 2B). Statistical comparison of CI values at ED_90_ showed that TNBC cell lines had significantly lower CI values than non-TNBC lines (*p* < 0.005). The strongest synergy was observed in SUM159 and MB-231 cells, with CI values of 0.541 and 0.657, respectively. Notably, both high (MB-231) and moderate (SUM159) SIK2 expression TNBC cells exhibited strong sensitivity to combined treatment, suggesting that the extent of pharmacologic response may not strictly correlated with basal SIK2 expression levels across TNBC cell lines. These results indicate that SIK2 inhibition markedly potentiates paclitaxel efficacy in TNBC, supporting a mechanistic link between SIK2 signaling and chemotherapy response.

### 3.3. GRN-300 Suppresses Clonogenic Growth and Potentiates Paclitaxel Activity in TNBC Cells

To evaluate the long-term proliferative consequences of SIK2 inhibition, we performed clonogenic assays using two representative TNBC cell lines: MB-231 and SUM159. Cells were seeded at low density and treated continuously with different concentrations of GRN-300 (0.5 μM, 1 μM, and 2 μM), and colonies were quantified after 10–14 days (Figure S2). GRN-300 alone reduced colony formation in a dose-dependent manner in both cell lines, demonstrating that sustained SIK2 inhibition impairs clonogenic survival. In SUM159 cells, significant inhibition was observed beginning at 1 μM (*p* < 0.005), whereas MB-231 cells showed greater sensitivity, with significant reduction detected at 0.5 μM (*p* < 0.01). To determine whether GRN-300 could potentiate the long-term cytotoxic effects of paclitaxel, we evaluated colony-forming ability following combined treatment with GRN-300 (1 μM) and paclitaxel (2 nM). In both MB-231 and SUM159 cells, the combination markedly reduced colony outgrowth compared with either monotherapy (Figure 2C). Notably, the combination nearly eliminated colony formation in SUM159 cells, mirroring the synergistic effects observed in short-term viability assays. These results demonstrate that GRN-300 not only inhibits clonogenic survival but also significantly enhances paclitaxel-mediated cytotoxicity in TNBC models.

### 3.4. Combination Therapy Demonstrated Enhanced Antitumor Efficacy in TNBC Xenograft Models

**Figure 3:**
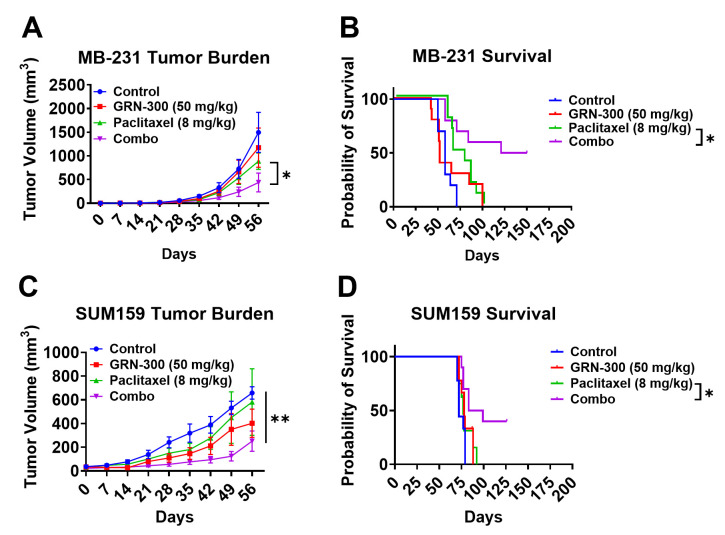
(**A**,**B**) MB-231 in vivo study. Tumor growth and survival were examined in female nu/nu mice that were injected orthotopically with 8.0 × 10^5^ MB-231 cells per mouse. Seven days after implantation, mice were randomized into four treatment groups (n = 10 per group): controls, paclitaxel, GRN-300, or the combination. (**C**,**D**) SUM159 in vivo study. Tumor growth and survival in female nu/nu mice that were injected orthotopically with 3.0 × 10^6^ SUM159 cells per mouse. The mice were randomized into treatment groups seven days after implantation (n = 10 per group). Statistical significance for tumor growth was determined using two-way ANOVA. Survival analyses were performed using Log-rank (Mantel–Cox) test. * *p* < 0.05, ** *p* < 0.01.

To determine whether the synergistic effects of GRN-300 and paclitaxel observed in cell culture translated to improved antitumor activity in vivo, we conducted two independent TNBC xenograft studies using MB-231 and SUM159 cells implanted into nu/nu mice. Animals were randomized to receive vehicle control, GRN-300 (50 mg/kg, five times per week, by gavage), paclitaxel (8 mg/kg, once weekly, ip), or combination therapy. In the MB-231 model, the combination produced significantly greater tumor growth inhibition than either single agent, leading to a marked improvement in overall survival (Figure 3A,B). A substantial proportion of mice receiving combination therapy achieved durable long-term survival, whereas animals in the monotherapy groups exhibited progressive tumor growth. Similarly, in the SUM159 xenograft model, single-agent GRN-300 or paclitaxel produced only modest antitumor effects. However, the combination therapy resulted in a robust and sustained reduction in tumor burden and significantly prolonged overall survival relative to all control groups (Figure 3C,D). Body weights remained stable throughout the treatment period across all groups, with no significant changes observed during GRN-300 and paclitaxel combination therapy. These in vivo findings confirm that SIK2 inhibition potentiates the antitumor efficacy of paclitaxel and supports the therapeutic potential of GRN-300 as a chemosensitizer in TNBC.

### 3.5. GRN-300 Promotes Paclitaxel-Induced G2/M Arrest and Apoptosis

**Figure 4:**
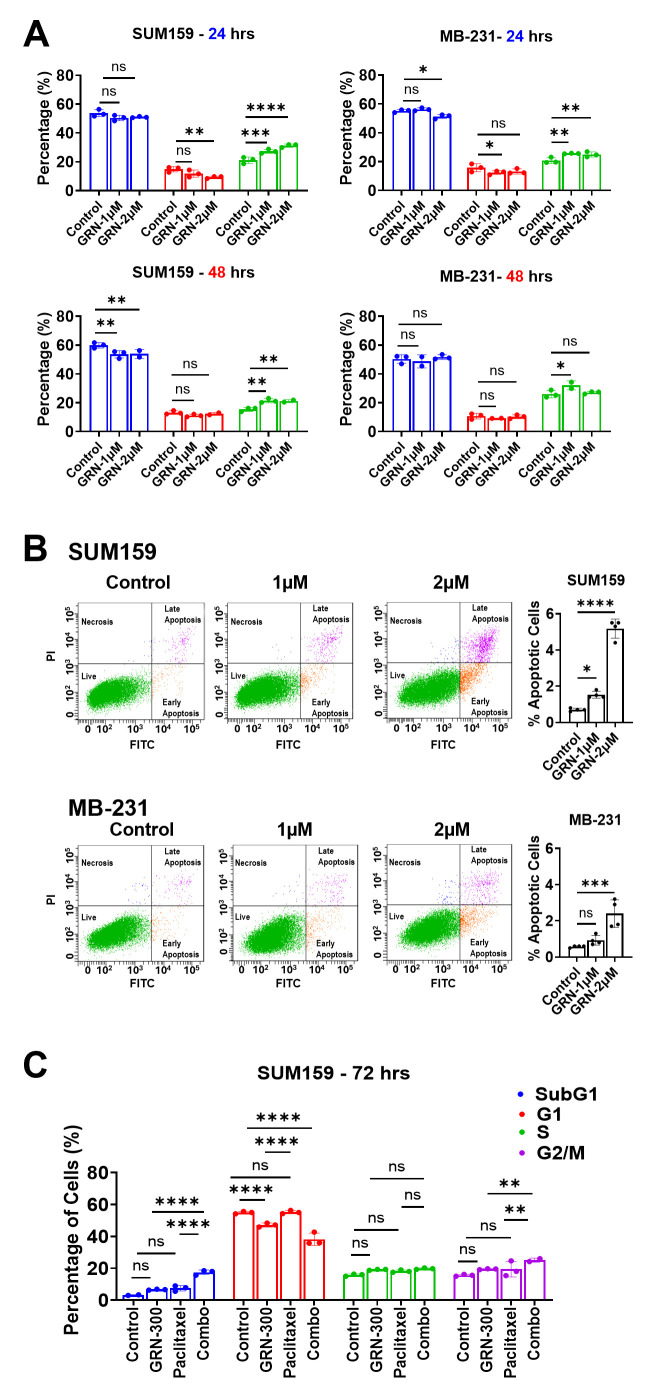
(**A**) Cell cycle analysis of breast cancer cells treated with GRN-300. MB-231 and SUM159 cell cycle distribution following GRN-300 treatment was assessed by PI/RNase staining. Triplicate samples for each treatment condition and timepoint were analyzed. Cell cycle data were collected using a Gallios analyzer. Population gating, single cell gating, and histogram analyses were performed using the FlowJo softwarev10.10.0. Statistical significance was determined by analyzing cell cycle phase percentages in GraphPad Prism 10 using two-way ANOVA. ns, *p* > 0.05, * *p* < 0.05, ** *p* < 0.01, *** *p* < 0.001, **** *p* < 0.0001. (**B**) Apoptosis analysis of breast cancer cells treated with GRN-300. SUM159 and MB-231 cells were treated with GRN-300 (1 µM or 2 μM) for 48 h, and apoptosis was assessed using Annexin V-FITC staining. Triplicate samples for each treatment condition were analyzed. Data were acquired using a Fortessa X-20 cytometer, and gating and histogram analyses were performed using the FlowJo software. Statistical significance was determined using the GraphPad Prism 10 program with one-way ANOVA analysis. ns, *p* > 0.05, * *p* < 0.05, *** *p* < 0.001, **** *p* < 0.0001. (**C**) Cell cycle analysis of breast cancer cells treated with the combination of GRN-300 and paclitaxel. SUM159 cells were treated with GRN-300 alone, paclitaxel alone, or the combination. Cell-cycle distribution was analyzed by PI/RNase staining. Triplicate samples for each treatment condition and time point were analyzed. Flow cytometry data were acquired using a Gallios analyzer. Gating and histogram analyses were performed using FlowJo software. Statistical significance was determined using the GraphPad Prism 10 program with two-way ANOVA. ns, *p* > 0.05, ** *p* < 0.01, **** *p* < 0.0001.

Because paclitaxel exerts its cytotoxic effects by stabilizing microtubules and inducing G2/M cell-cycle arrest, we next examined whether SIK2 inhibition enhances this checkpoint blockade. Treatment of SUM159 and MB-231 cells with GRN-300 alone produced a significant increase in the proportion of cells accumulating in the G2/M phase at 24 h, indicating that SIK2 activity contributes proper cell-cycle progression (Figure 4A). This effect was dose-dependent and remained evident at 48 h in both cell lines, suggesting a sustained disruption of mitotic transit. To determine whether SIK2 inhibition also induces apoptosis, we performed Annexin V-FITC staining. GRN-300 treatment increased early apoptotic populations in a dose-dependent manner in both SUM159 and MB-231 cells (Figure 4B). At 2 μM, GRN-300 increased the percentage of Annexin V-positive cells by approximately 4% relative to untreated controls, indicating activation of apoptotic signaling in addition to G2/M arrest. We next assessed whether GRN-300 enhances the cell-cycle effects of paclitaxel. At extended time points (72 h), combination treatment resulted in a significantly higher fraction of cells arrested in G2/M compared with either single agent or control (Figure 4C), demonstrating that SIK2 inhibition reinforces paclitaxel-mediated mitotic blockade. Consistent with these findings, we also observed a marked increase in the Sub-G1 population, an indicator of DNA fragmentation and apoptotic cell death, was observed, in both single-agent and combination groups at 72 h. At earlier points (24 h and 48 h), GRN-300 monotherapy produced the most pronounced G2/M accumulation, while a modest increase in the Sub-G1 fraction was detected across all treatment conditions (Figure S3). Together, these results demonstrate that GRN-300 induces G2/M arrest and apoptosis as a single agent and cooperates with paclitaxel to reinforce mitotic arrest and promote apoptotic cell death in TNBC models.

### 3.6. SIK2 Inhibition Disrupts Mitotic Regulatory Signaling and Enhances Apoptotic Responses

**Figure 5:**
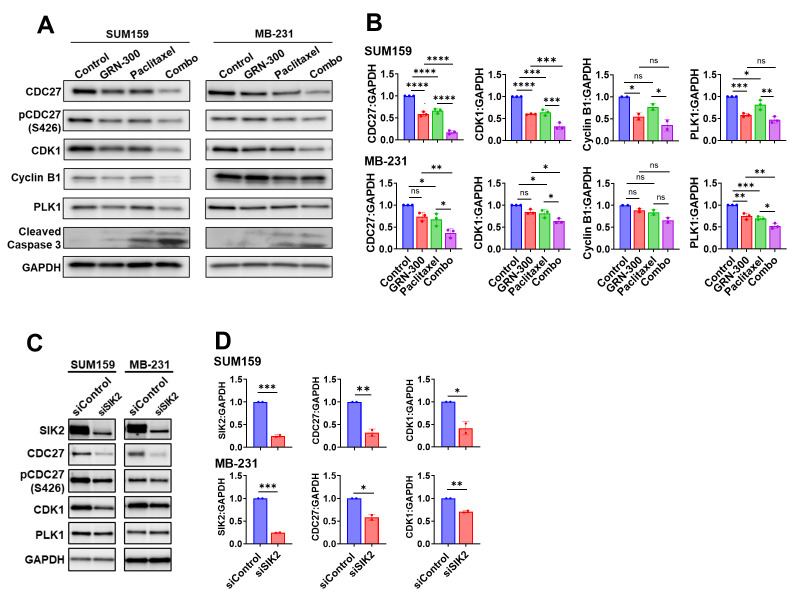
(**A**,**B**) Western blot analysis of mitotic and apoptotic protein markers in breast cancer cells. SUM159 and MB-231 cells were treated with GRN-300 (1 μM) and/or Paclitaxel (2 nM) for 48 h. Protein expression was analyzed by Western blot using the indicated antibodies. APC/C mitotic pathway-related proteins and apoptotic markers were evaluated. (**C**,**D**) Western blot analysis of mitotic protein markers following SIK2 knockdown. SUM159 and MB-231 cells were transfected with control siRNA (siControl) and siSIK2 at a final concentration of 35 nM for 72 h. SIK2 knockdown efficiency was confirmed by Western blot analysis. Western blot images were acquired using a Bio-Rad ChemiDoc imaging system. Experiments were independently repeated twice, and band intensities were quantified using ImageJ software. Statistic analysis was conducted in GraphPad Prism using one-way ANOVA or student *t*-test. ns *p* > 0.05, * *p* < 0.05, ** *p* < 0.01, *** *p* < 0.001, **** *p* < 0.0001.

To further elucidate the molecular mechanisms underlying GRN-300-induced cell-cycle arrest, we examined the expression of key mitotic regulatory proteins following SIK2 inhibition. Consistent with its role as a selective SIK2 inhibitor, GRN-300 treatment markedly reduced SIK2 protein levels. This reduction was accompanied by decreased expression of several critical mitotic regulators, including CDC27, phospho-CDC27 (pCDC27), CDK1, and PLK1 (Figure 5A,B). Because these proteins play central roles in anaphase-promoting complex/cyclosome (APC/C) activation and mitotic progression, their coordinated downregulation suggests that GRN-300 disrupts the core mitotic signaling pathway. To confirm that these effects were specifically mediated through SIK2 inhibition, we performed siRNA-mediated knockdown of SIK2. Similar to GRN-300 treatment, SIK2 depletion resulted in substantial reductions in CDC27, CDK1, and PLK1 expression (Figure 5C,D), supporting a role for SIK2 in maintaining mitotic regulatory signaling. In addition, GRN-300 treatment significantly decreased cyclin B1 expression (Figure 5A,B), a key component of the CDK1-cyclin B1 complex required for mitotic entry and progression. The concurrent reduction in cyclin B1 and APC/C-associated regulators further supports the conclusion that SIK2 inhibition perturbs multiple components of the mitotic machinery, contributing to G2/M arrest. Finally, consistent with the increased apoptosis detected by cell-cycle analyses, combination treatment with GRN-300 and paclitaxel markedly elevated cleaved caspase-3 levels compared with either agent alone (Figure 5B), indicating enhanced induction of apoptotic cell death. Together, these findings demonstrate that SIK2 inhibition disrupts mitotic regulatory pathways and enhances chemotherapy-induced apoptosis.

### 3.7. CDC27 Overexpression Partially Restores Mitotic Signaling Following SIK2 Inhibition

**Figure 6:**
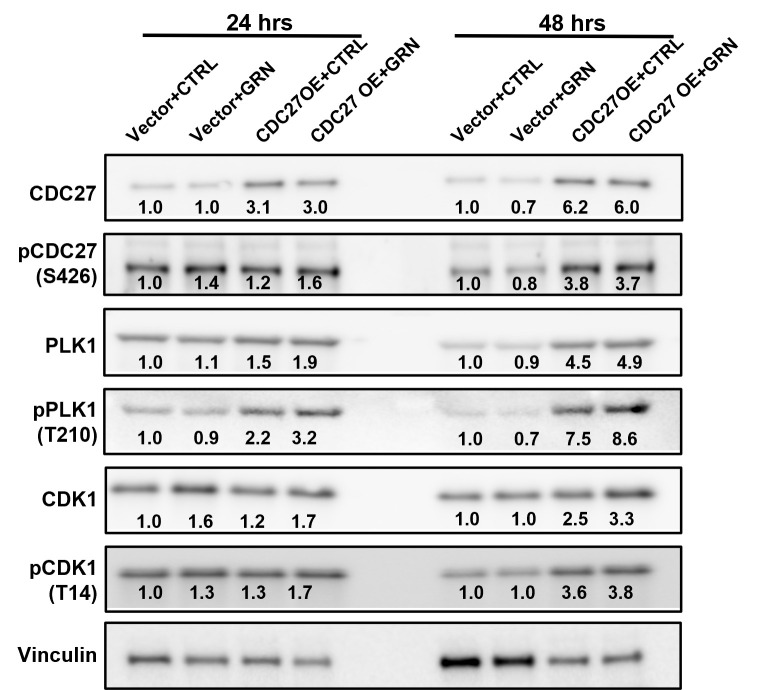
CDC27 overexpression in TNBC cells. SUM159 cells were transduced with control lentivirus (pLOC) or pLOC-CDC27 to induce CDC27 expression. Cells were then treated with GRN-300 (2 μM) for 24 h or 48 h. Protein expression levels of total and phosphorylated CDC27, PLK1, and CDK1 were evaluated by Western blot analysis. Band intensity was quantified using ImageJ software, normalized to vinculin loading controls, and fold changes are shown beneath each band.

To further explore the role of CDC27 in mediating the effects of SIK2 inhibition, we generated a lentiviral CDC27-overexpressing SUM159 stable cell line. Successful overexpression of CDC27 was confirmed by immunoblot analysis (Figure 6). Notably, forced CDC27 expression attenuated the suppressive effects of the SIK2 inhibitor GRN-300 on key mitotic regulators, as evidenced by the restoration of phospho-PLK1 and phospho-CDK1 levels that were otherwise reduced upon GRN-300 treatment (Figure 6). These findings indicate that CDC27 acts downstream of SIK2 and contributes to the maintenance of CDK1 and PLK1 signaling during mitotic progression. Collectively, these results support a model in which SIK2 regulates CDC27-dependent APC/C mitotic signaling, and disruption of this pathway by GRN-300 impairs activation of critical mitotic kinases.

## 4. Discussion

This study demonstrates the therapeutic potential of combining GRN-300, a novel SIK2 inhibitor with paclitaxel for treating Triple-Negative Breast Cancer. Our data indicated that SIK2 was expressed in 88% of TNBC clinical specimens and in eight of nine TNBC cell lines, supporting the relevance of SIK2 as a therapeutic target in this breast cancer subtype. Given the limited targeted treatment options currently available for TNBC, these findings provide a strong rationale for further investigation of SIK2-directed therapeutic strategies.

Paclitaxel exerts its antitumor activity through stabilization of microtubules, resulting in prolonged mitotic arrest through activation of the spindle assembly checkpoint (SAC) [7,24]. Sustained SAC activation subsequently suppresses APC/C activation, a critical regulator required for mitotic exit [25,26,27]. Building on this mechanistic framework, we demonstrated that GRN-300 significantly enhanced sensitivity in TNBC models. Combination index analysis revealed synergistic interactions (CI < 1) in eight of nine TNBC cell lines tested, with the most pronounced synergy observed in SUM159 and MB-231 cell lines. In contrast, non-TNBC breast cancer cell lines showed limited or no enhancement with combination treatment, suggesting that the therapeutic effect of GRN-300 may be more pronounced in TNBC models. Importantly, the enhanced antitumor activity of the combination was validated in two in orthotopic xenograft models. Combination treatment produced greater tumor growth inhibition than either monotherapy alone and improved overall survival in both the MB-231 and SUM159 models, supporting the potential translational relevance of this therapeutic strategy.

To investigate the mechanisms underlying these effects, we examined the impact of SIK2 inhibition on cell-cycle progression and mitotic signaling. Previous work from our laboratory demonstrated that SIK2 regulates centrosome separation and mitotic progression in ovarian cancer models [17]. Consistent with these findings, GRN-300 treatment induced significant accumulation of TNBC cells in the G2/M phase of the cell cycle, indicating disruption of mitotic progression. This effect was particularly evident in the highly responsive MB-231 and SUM159 in the G2/M phase of the cell cycle, indicating disruption of mitotic progression. This effect was particularly evident in the highly responsive MB-231 and SUM159 cell lines and was sustained at both 24 h and 48 h time points in both cell lines. GRN-300 treatment also induced apoptosis, as demonstrated by increased Annexin V positivity and elevated cleaved caspase-3 expression. When combined with paclitaxel, an even greater proportion of cells accumulated in the G2/M phase, accompanied by increased Sub-G1 populations, suggesting enhanced apoptotic cell death following prolonged mitotic arrest. Together, these findings support a model in which SIK2 inhibition enhances paclitaxel-induced mitotic stress and promotes apoptotic cell death in TNBC cells.

Our molecular analysis further demonstrated that SIK2 inhibition disrupts key components of the APC/C regulatory network. GRN-300 treatment reduced expression of CDC27 and phospho-CDC27, as well as CDK1, PLK1, and cyclin B1, all of which are essential regulators of mitotic progression and APC/C activation [28,29]. Similar findings were observed following siRNA-mediated SIK2 knockdown, supporting the specificity of these effects. CDC27, also known as APC3, is a core APC/C subunit that regulates ubiquitination and degradation of key mitotic substrates, including securin and cyclin B1 [30]. Proper APC/C activation requires coordinated phosphorylation events mediated by CDK1 and PLK1 [25,31,32,33]. Therefore, the coordinated suppression of CDC27, CDK1, PLK1, and cyclin B1 following SIK2 inhibition suggests broad disruption of mitotic regulatory signaling. In addition, restoration of phospho-CDK1 and phospho-PLK1 expression following CDC27 overexpression further supports the functional importance of the SIK2-CDC27 signaling axis in maintaining mitotic progression.

Several limitations of this study should be acknowledged. Although our findings support a role for SIK2 in regulating APC/C-associated mitotic signaling, the precise molecular relationship between SIK2 and CDC27 remains incompletely defined and warrants further investigation. We did not perform CDC27 overexpression rescue experiments to directly test whether CDC27 can reverse the effect of the GRN-300/paclitaxel combination on cell viability, clonogenic survival, cell-cycle arrest, or apoptosis, which would provide more definitive causal evidence and represents an important direction for future studies. In addition, TNBC is a highly heterogeneous disease, and the impact of tumor heterogeneity on sensitivity to SIK2 inhibition was not extensively explored in this study. Future studies using a broader range of TNBC models and molecular subtypes will be important to better define determinants of therapeutic response. Furthermore, although we observed high SIK2 expressions in TNBC specimens and demonstrated that GRN-300 treatment affected cell growth, cell-cycle progression, and apoptosis, this study was not designed to directly establish whether SIK2 expression levels correlate with these phenotypic responses. Additional mechanistic studies will therefore be needed to determine whether SIK2 expression or APC/C pathway activity may serve as predictive biomarkers for sensitivity to SIK2-targeted therapies.

## 5. Conclusions

In summary, our finding demonstrates that SIK2 inhibition with GRN-300 enhances the antitumor activity of paclitaxel in TNBC models through disruption of mitotic regulatory signaling and promotion of apoptotic cell death. These results provide preclinical evidence supporting further development of GRN-300 in combination with paclitaxel as a potential therapeutic strategy for patients with TNBC.

## Data Availability

The data in the study generated by the authors are included in the article. Further inquiries can be directed to the corresponding author.

## Supplementary Materials

The following supporting information can be downloaded at: https://www.mdpi.com/article/10.3390/cancers18111843/s1, Table S1: Additional cell line information; Figure S1: GRN-300 and paclitaxel combination treatment in non-TNBC cell lines; Figure S2: GRN-300 treatment in TNBC cell lines; and Figure S3: Effects of SIK2 inhibition on cell cycle arrest and apoptosis. Uncropped WB images for revision.
