# Supplementary material for: Targeting SIK2 with GRN-300 Potentiates Paclitaxel Efficacy in Triple-Negative Breast Cancer
Source: Cancers (Basel). 2026 Jun 4;18(11):1843. doi: 10.3390/cancers18111843 (PMC13256359; doi:10.3390/cancers18111843)

Table S1. Additional cell line information

Cell line	Database	RRID	Genetic alterations
MDA-MB-231	Cellosaurus	CVCL_0062	TP53 mutation,<br>CDKN2A homozygous deletion,<br>CDKN2B homozygous deletion,<br>BRAF mutation,<br>KRAS mutation,<br>TERT
MDA-MB-468	Cellosaurus	CVCL_0419	PTEN mutation,<br>RB1 mutation,<br>TP53 mutation
MDA-MB-436	Cellosaurus	CVCL_0623	FHIT homozygous deletion,<br>BRCA1 mutation,<br>RB1 mutation,<br>TP53 mutation
SUM159	Cellosaurus	CVCL_5423	HRAS mutation,<br>PIK3CA mutation,<br>TP53 mutation
HCC1143	Cellosaurus	CVCL_1245	TP53 mutation
HCC1395	Cellosaurus	CVCL_1249	TP53 mutation
HCC1428	Cellosaurus	CVCL_1252	Gene fusion
HCC1569	Cellosaurus	CVCL_1255	BRCA2 mutation,<br>TP53 mutation,<br>PTEN mutation,<br>FHIT mutation
HCC1937	Cellosaurus	CVCL_0290	PTEN deletion,<br>BRCA1 mutation,<br>RB1 mutation,<br>TP53 mutation,<br>TMPRSS2 mutation
HCC1954	Cellosaurus	CVCL_1259	PIK3CA mutation,<br>TP53 mutation,<br>Gene fusion
HCC202	Cellosaurus	CVCL_2062	PIK3CA mutation,<br>TP53 mutation
HCC38	Cellosaurus	CVCL_1267	TP53 mutation
HCC70	Cellosaurus	CVCL_1270	TP53 mutation
BT-474	Cellosaurus	CVCL_0179	BRCA2 mutation,<br>TP53 mutation,<br>PIK3CA mutation,<br>MAPK1 mutation,<br>HNF1A mutation,<br>Multiple gene fusion
T47D	Cellosaurus	CVCL_0553	TP53 mutation,<br>PIK3CA mutation
MCF7	Cellosaurus	CVCL_0031	TP53 mutation,<br>PIK3CA mutation,

			GATA3 mutation,<br>CDKN2A mutation
SKBr3	Cellosaurus	CVCL_0033	TP53 mutation,<br>CDH1 homozygous deletion
ZR75-1	Cellosaurus	CVCL_0588	HRAS mutation,<br>PTEN mutation
Cal51	Cellosaurus	CVCL_1110	PIK3CA mutation,<br>RRAS3 mutation
KPL-4			PIK3CA mutation reported by Huw L-Y et al. https://doi.org/10.1038/oncsis.2013.46

Figure S1. GRN-300 and paclitaxel combination treatment in non-TNBC cell lines. Paclitaxel combination treatment with GRN-300. Dose-response curves were generated using GraphPad Prism 10. Each cell line had its Paclitaxel IC50 independently calculated using GraphPad Prism 10. Paclitaxel IC50 was used for the combination treatment with GRN-300.

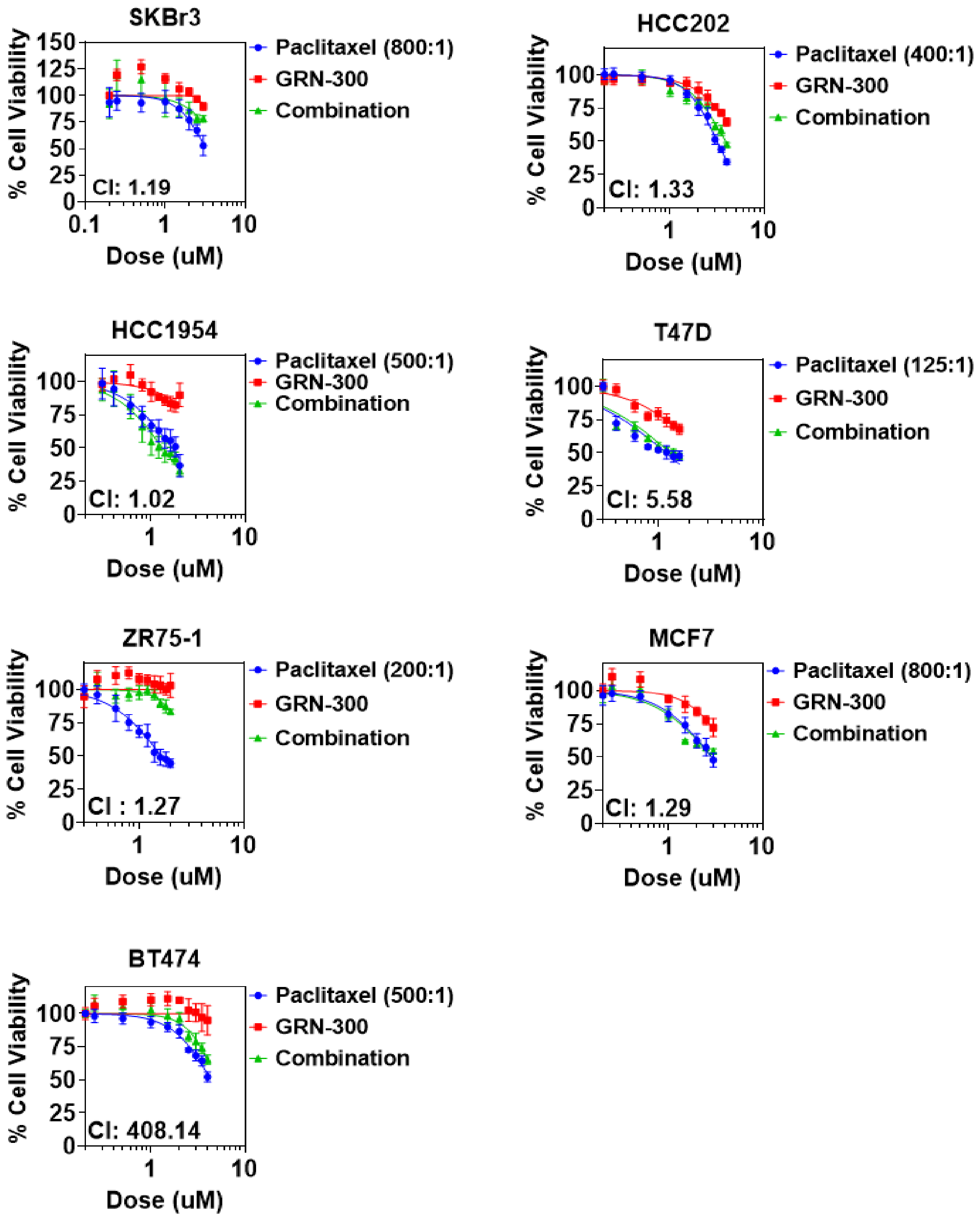

Figure S2. GRN-300 treatment in TNBC cell lines. Clonogenic assay of MB-231 and SUM159 cells lines treated with GRN-300 alone in different concentrations as indicated. Colony area was quantified using ImageJ, and statistical significance was determined by one-way ANOVA with GraphPad Prism 10. ** P < 0.01; **** P < 0.0001.

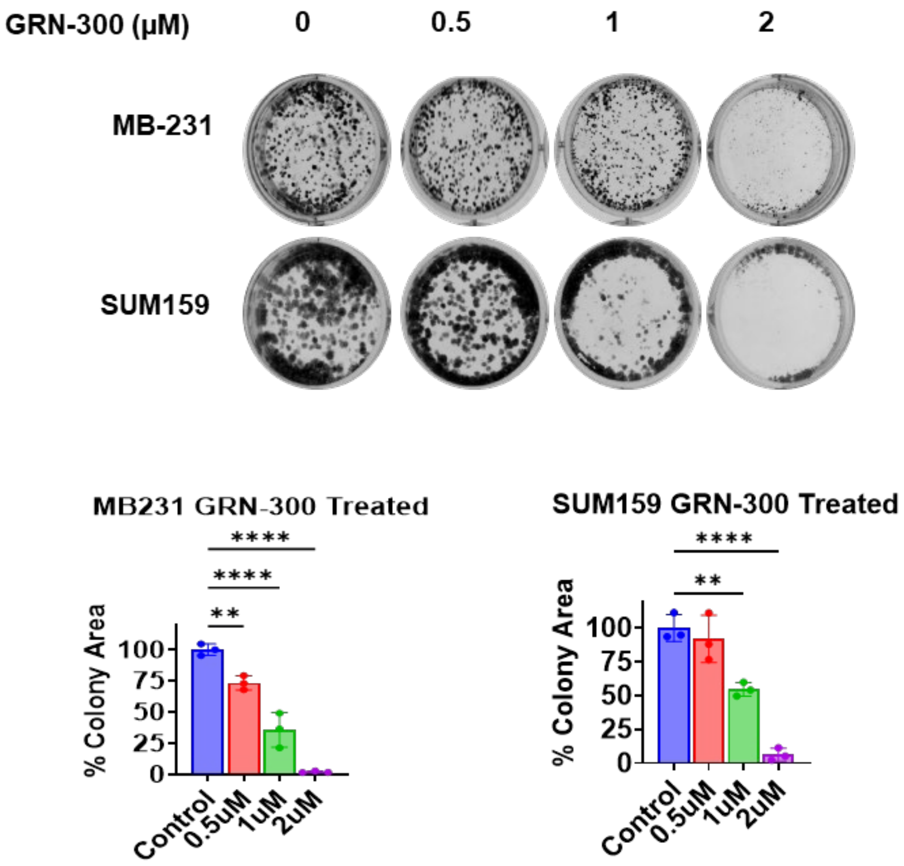

Figure S3. Effects of SIK2 inhibition on cell cycle arrest and apoptosis. Cell cycle analysis of SUM159 cells treated with GRN-300, paclitaxel, or the combination for indicated hours. Triplicate samples were collected for each condition. Data were acquired on a Gallios analyzer and analyzed in FlowJo. Statistical significance was determined by two-way ANOVA with GraphPad Prism 10. NS, $P > 0.05$; *$P < 0.05$; **$P < 0.01$; ****$P < 0.0001$.

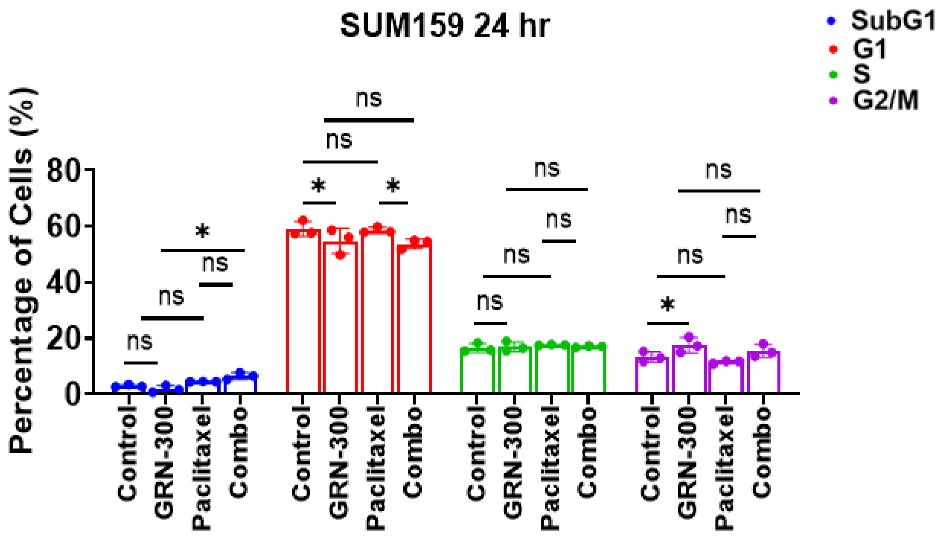

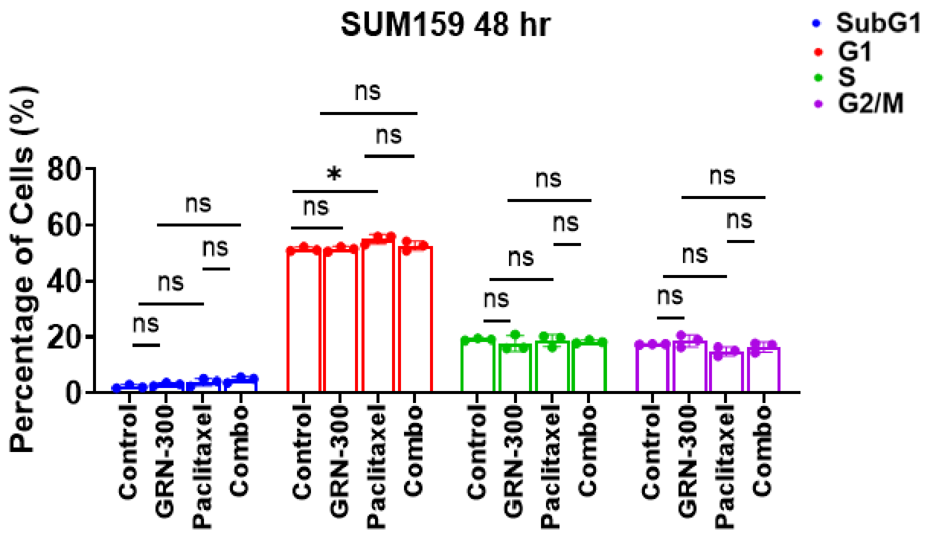

Uncropped WB images for revision

Figure 1B.

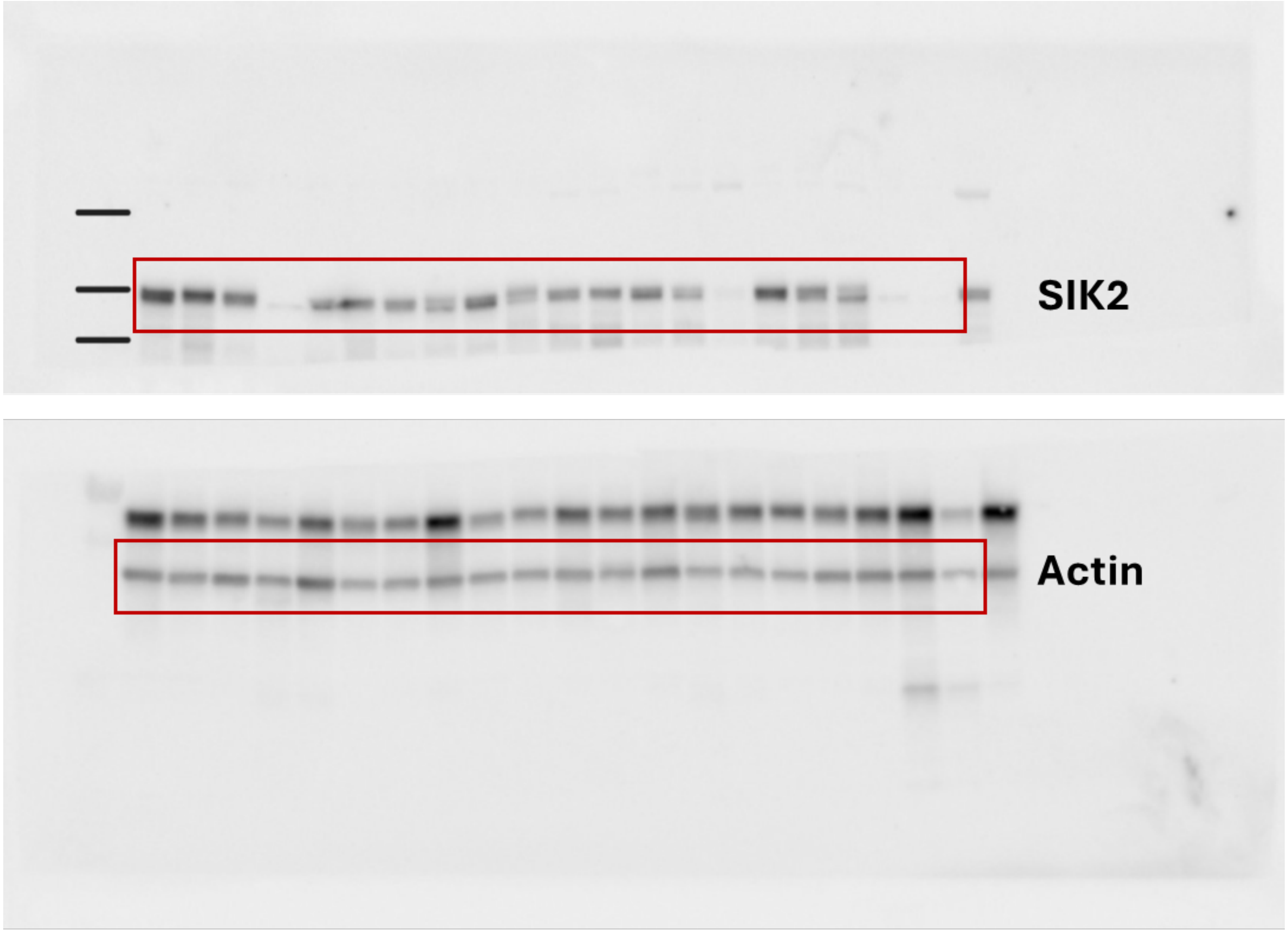

Figure 5A

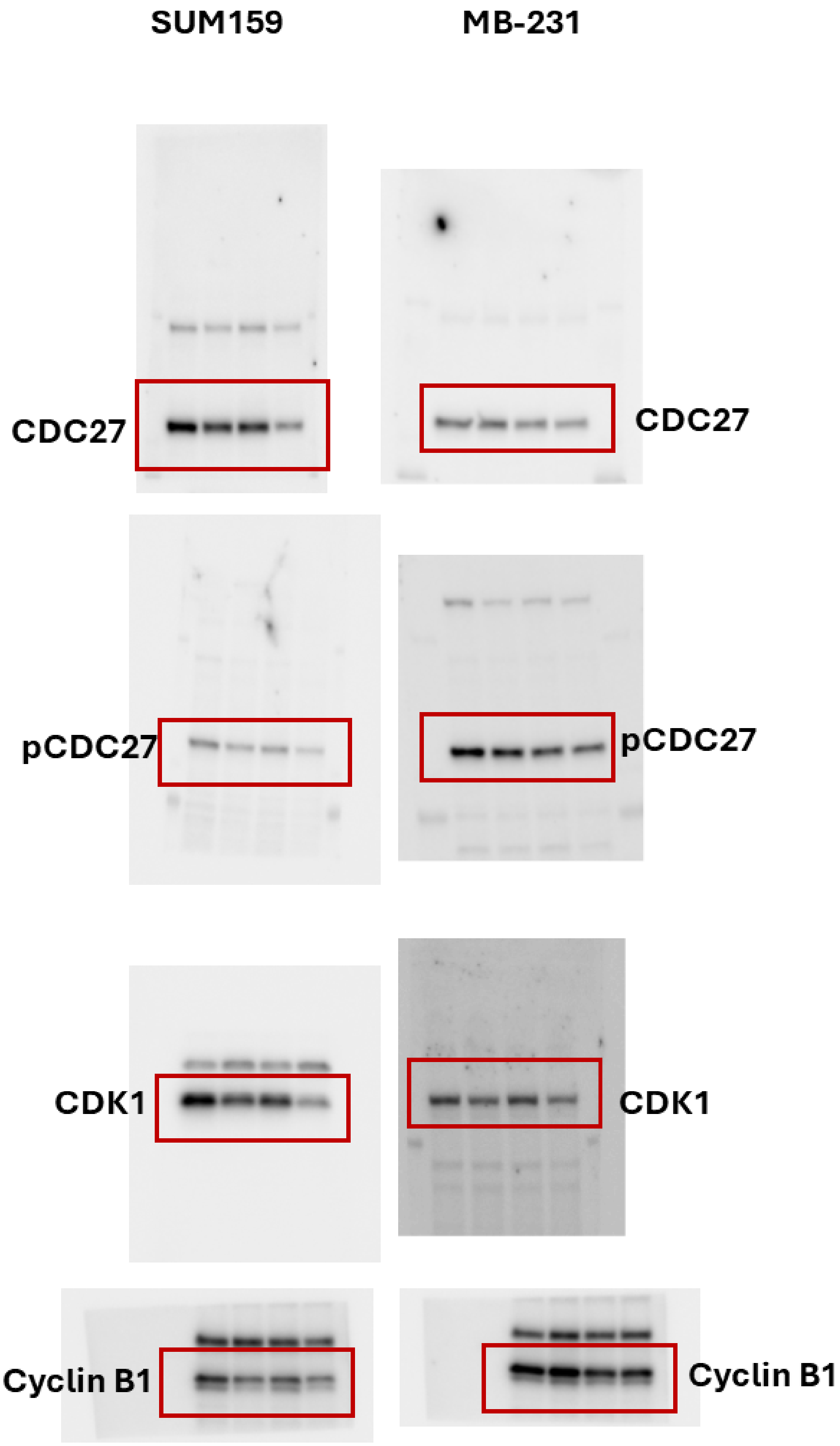

Experiment A29, A30 and A31 folder

Figure 5A. continued

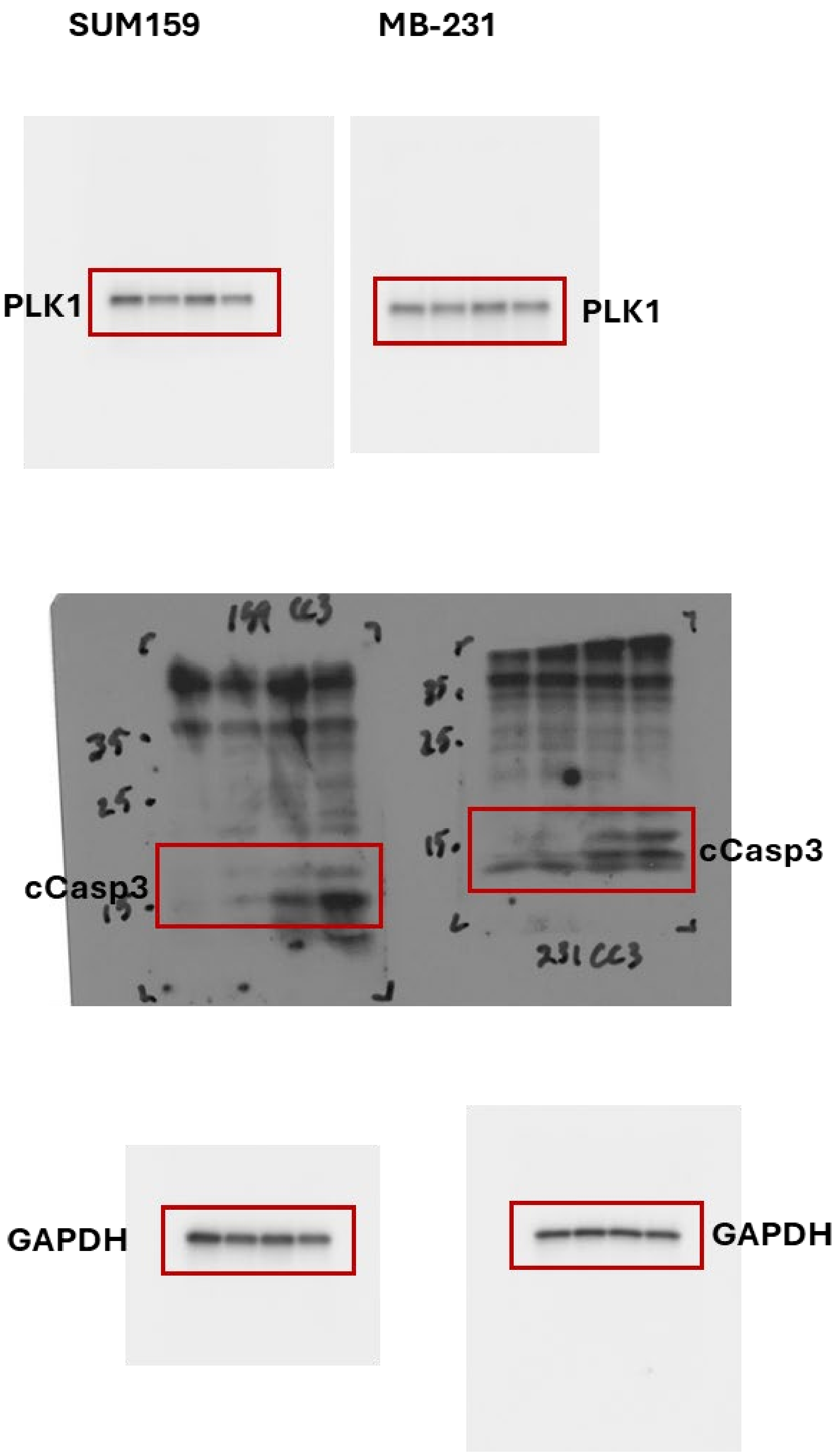

Figure 5C.

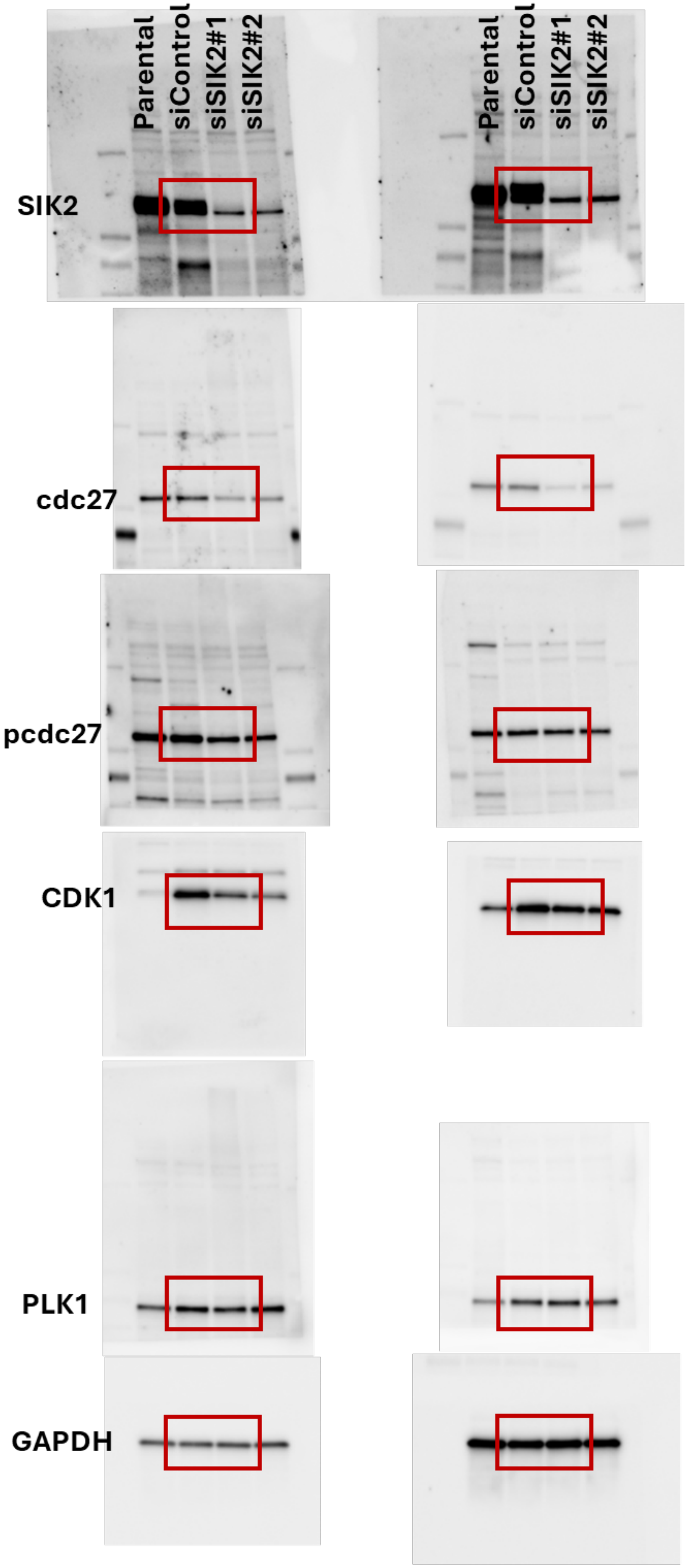

Parental
siControl
siSIK2#1
siSIK2#2
Parental
siControl
siSIK2#1
siSIK2#2
SIK2
cdc27
pcdc27
CDK1
PLK1
GAPDH

Figure 6.

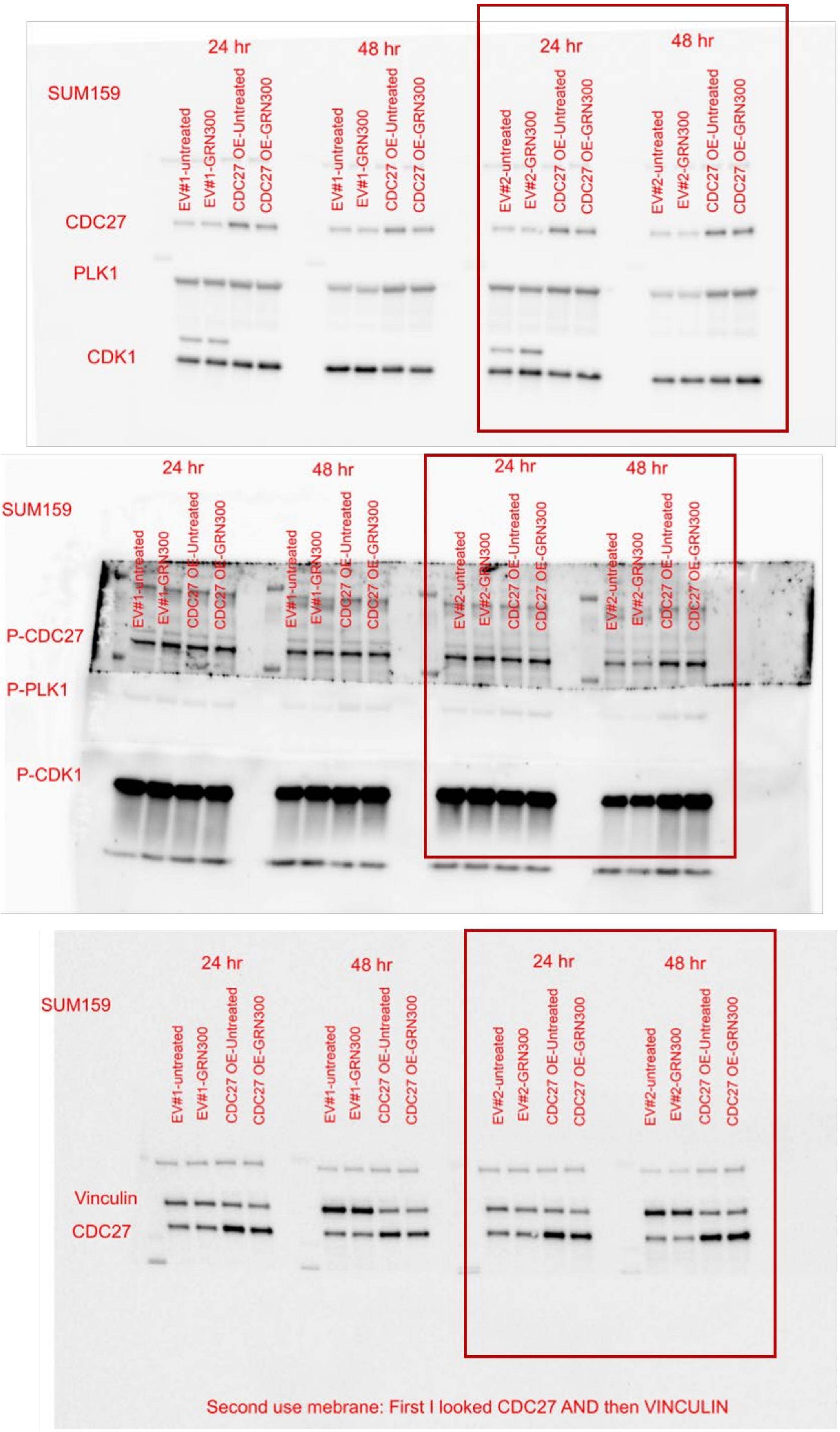
SUM159
24 hr
48 hr
24 hr
48 hr
EV#1-untreated
EV#1-GRN300
CDC27 OE-Untreated
CDC27 OE-GRN300
EV#2-untreated
EV#2-GRN300
CDC27 OE-Untreated
CDC27 OE-GRN300
CDC27
PLK1
CDK1
SUM159
24 hr
48 hr
24 hr
48 hr
EV#1-untreated
EV#1-GRN300
CDC27 OE-Untreated
CDC27 OE-GRN300
EV#2-untreated
EV#2-GRN300
CDC27 OE-Untreated
CDC27 OE-GRN300
P-CDC27
P-PLK1
P-CDK1
SUM159
24 hr
48 hr
24 hr
48 hr
EV#1-untreated
EV#1-GRN300
CDC27 OE-Untreated
CDC27 OE-GRN300
EV#2-untreated
EV#2-GRN300
CDC27 OE-Untreated
CDC27 OE-GRN300
Vinculin
CDC27
Second use mebrane: First I looked CDC27 AND then VINCULIN

Figure 6. continued

Long exposure

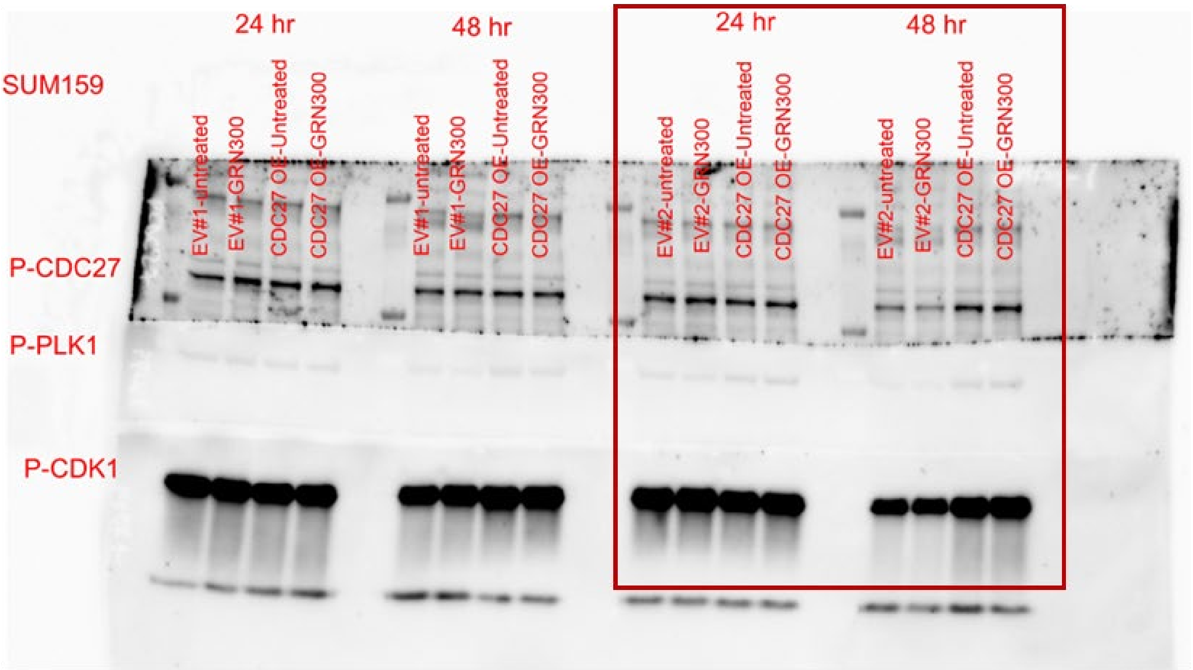

Short exposure

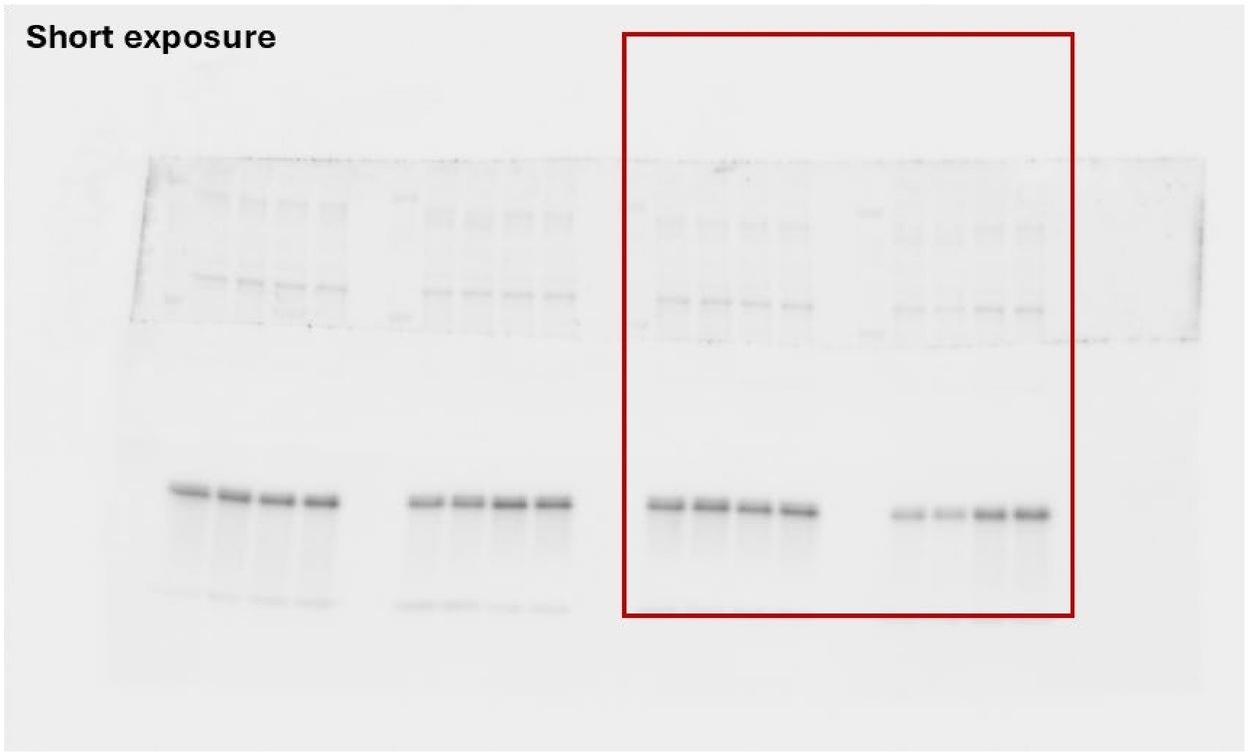

Supplement: Supplementary file 1 [file cancers-18-01843-s001.zip › cancers-4315680-supplementary.pdf]
